# Anti-NY-ESO-1 autoantibody may be a tumor marker for intrahepatic cholangiocarcinoma

**DOI:** 10.18632/oncotarget.22464

**Published:** 2017-11-15

**Authors:** Zhen Zhang, Fan-Fan Li, Ming-Dian Lu, Shang-Xin Zhang, Yong-Xiang Li

**Affiliations:** ^1^ Department of Gastrointestinal Surgery, The First Affiliated Hospital of Anhui Medical University, Hefei, China; ^2^ Department of Oncology, The Second Affiliated Hospital of Anhui Medical University, Hefei, China

**Keywords:** NY-ESO-1, antibody, intrahepatic cholangiocarcinoma, prognosis

## Abstract

Anti-NY-ESO-1 antibody is observed in a multitude of malignancies. This study was aimed to evaluate the expression of serum anti-NY-ESO-1 antibodies and its prognostic value in intrahepatic cholangiocarcinoma. A total of 103 patients with intrahepatic cholangiocarcinoma were enrolled in the study. Enzyme-linked immunosorbent assay (ELISA) was performed to detect the serum level of anti-NY-ESO-1 antibody. Western blotting was performed to assess the NY-ESO-1 expression in tumor and adjacent tissues. The serum NY-ESO-1 antibody was detected in 18.4% of patients with intrahepatic cholangiocarcinoma, a value that was significantly higher than that in patients with chronic Hepatitis B. Serum NY-ESO-1 antibody was positively correlated with tumor differentiation, lymphatic metastasis, cTNM stage and abdominal pain. Finally, there was a higher cumulative survival rate in patients with serum NY-ESO-1 positivity than in those with serum NY-ESO-1 negativity among the patients with stage III + IV. Our data uncovered that NY-ESO-1 antibody might be a helpful tumor marker and prognostic predictor in intrahepatic cholangiocarcinoma.

## INTRODUCTION

Cholangiocarcinoma, a heterogeneous entity of malignancy derived from epithelial biliary cells, is classified into intrahepatic (iCCA), perihilar (pCCA) or distal cholangiocarcinomas according to different anatomical locations [1]. Cholangiocarcinoma is rare malignancy and accounts for 5% to 10% of the malignant tumors [2]. Since the high-level resistance to conventional antitumor agents and poor clinical outcome, surgical treatment is the preferred option and only curative treatment for all subtypes. However, the surgical treatment is hampered by the high incidence of recurrence and metastasis and a low 5-year overall survival rate [3]. Therefore, the identification of sensitive prognostic and predictive markers is of great clinical importance to the improvement of the curative rate and survival rate of the patients.

The analysis of serum tumor markers is used for screening, predicting response, and postoperative surveillance of malignancies including hepatobiliary cancer [4]. The combined determination of several nonspecific tumor markers, including cancer antigen (CA) 72-4, CA125, CA19-9 and carcinoembryonic antigen (CEA), is an effective method to monitor the treatment efficacy and predict tumor recurrence [5]. Previous studies have presented that post-operation monitoring of CEA and/or CA19-9 is useful to predict the recurrence or evaluate the response of patients with iCCA, especially patients with high preoperative levels of these markers [6, 7]. However, these aforementioned markers are non-organ-specific and merely available in disease activity evaluation and treatment efficacy monitoring, but not in the diagnosis of iCCA [8, 9].

The cancer/testis (CT) antigen of NY-ESO-1 was initially defined during the identification of immunogenic tumor antigens in the case of esophageal squamous cell carcinoma using the serological analysis of recombinant cDNA expression libraries (SEREX) [10, 11]. Further studies have demonstrated that humoral immunity to NY-ESO-1, with the spontaneous production of NY-ESO-1 antibody, is clearly antigen driven because it is reduced with tumor resection or histopathological regression [12]. The frequency of NY-ESO-1 antibody was 9.4% in melanoma patients, 12.5% in ovarian cancer patients, 3.8–52% in prostate cancer patients, and 9–23% in lung cancer patients; on the other hand, NY-ESO-1 antibody has not been found in non-cancerous patients or healthy control subjects [13–16]. These findings revealed that NY-ESO-1 antibody may be a promising candidate for the cancer diagnosis and immunotherapy of various cancers.

However, there were rare reports focused on the expression of NY-ESO-1 gene or antigen in iCCA [17, 18] and no study was conducted for the correlation between serum antibody against NY-ESO-1 and survival of patients with cholangiocarcinoma especially iCCA. The aim of this study was to investigate the expression of NY-ESO-1 antibody and its association with different clinicopathological features and the survival rate of patients with iCCA.

## RESULTS

### Clinical profile of enrolled subjects and NY-ESO-1 antibody levels

The clinical characteristics and serum level of NY-ESO-1 antibody of the subjects are demonstrated in Table 1. A total of 103 patients with iCCA and 108 CHB patients matched for age and sex were recruited in this study. In patients with iCCA and CHB, the mean ages were 65.4 ± 13.4 and 62.7 ± 15.2 years, respectively; the percentages of female patients were 67.0% (69/103) and 64.8% (70/108), respectively. There was significant difference between the two populations in AFP level (36.4 ± 13.1 ng/ml vs. 7.1 ± 20.5 ng/ml), total bilirubin (T-Bil) (124.9 ± 35.7 μmol/L vs. 38.3 ± 12.2 μmol/L), and direct bilirubin (D-Bil) (23.7 ± 36.2 μmol/L vs. 15.6 ± 11.1 μmol/L) (all *p <* 0.001), respectively. Most importantly, the positivity of NY-ESO-1 antibody was 18.4% (19/103) in patients with iCCA, which was significantly higher than that in patients with CHB (3.7%, 4/108) (*p <* 0.001). The median duration of follow-up was 24 months (range, 1–36 months).

**Table 1:** Characteristics of the subjects and expression of anti-NY-ESO-1 antibody

	ICC (*n* = 103)	CHB (*n* = 108)	*P*
Age (year)	65.4 ± 13.4	62.7 ± 15.2	0.67
Gender (female, %)	69 (67.0)	70 (64.8)	0.28
Smoking (*n*, %)	54 (52.4)	61 (56.5)	0.49
Alcohol drinking (*n*, %)	46 (44.7)	58 (53.7)	0.07
Anti-NY-ESO-1 (*n*, %)	19 (18.4)	4 (3.7)	<0.001
Laboratory parameters			
Anti-HBV (+) (*n*, %)	49 (47.6)	56 (51.9)	0.49
AFP (ng/ml)	36.4 ± 13.1	7.1 ± 20.5	<0.001
T-Bil (μmol/L)	124.9 ± 35.7	38.3 ± 12.2	<0.001
D-Bil (μmol/L)	23.7 ± 36.2	15.6 ± 11.1	<0.001
AST (U/L)	57.2 ± 11.2	66.2 ± 15.2	0.81
ALT (U/L)	54.2 ± 20.3	56.1 ± 37.3	0.79
GGT (U/L)	165.3 ± 435.2	162.2 ± 31.5	0.45

ICC: Intrahepatic cholangiocarcinoma; CHB: Chronic Hepatitis B.

### Serum NY-ESO-1 antibody and pathological features of iCCA

There were 21.1% (4/19) of well differentiated and 78.9% (15/19) of poorly differentiated iCCAs in serum NY-ESO-1 antibody-positive patients, leading to a significantly different distribution of histological grade between iCCA patients with different NY-ESO-1 humoral immune responses (*p =* 0.01). However, there was no significant difference in tumor size (*p =* 0.60), portal invasion (*p* = 0.06) and liver cirrhosis (*p =* 0.82) between NY-ESO-1 antibody-positive and -negative patients (*p* > 0.05). Furthermore, the relationship between NY-ESO-1 antibody positivity and lymphatic metastasis and clinical TNM (cTNM) stage of iCCA were analyzed. The results demonstrated that a significant difference in lymphatic metastasis and cTNM stage was found between the positive expression of serum NY-ESO-1 and cTNM stage (*p =* 0.04 and *p =* 0.03, respectively) (Table 2).

**Table 2:** The relationship between positivity of anti-NY-ESO-1 antibodies and pathological characteristics

Characteristic	Anti-NY-ESO-1 (+) (*n* = 19)	Anti-NY-ESO-1 (–) (*n* = 84)	*P*
Differentiation (*n*, %)			
Well differentiation	4 (21.1)	44 (52.4)	0.01
Poorly differentiation	15 (78.9)	40 (47.6)	
Tumor size (*n*, %)			
≤5 cm	6 (31.6)	32 (38.1)	0.60
>5 cm	13 (68.4)	52 (61.9)	
Portal invasion			
Yes	12 (63.2)	33 (39.3)	0.06
No	7 (36.8)	51 (60.7)	
Lymphatic metastasis (*n*, %)			
Yes	11 (57.9)	27 (32.1)	0.04
No	8 (42.1)	57 (67.9)	
Liver cirrhosis			
Yes	9 (47.4)	39 (46.4)	0.82
No	10 (52.6)	45 (43.6)	
cTNM stage (*n*, %)			
I + II	6 (31.6)	50 (59.5)	0.03
III + IV	13 (68.4)	34 (40.5)	

### Expression of NY-ESO-1 protein in iCCA tissue

**Figure 1:**
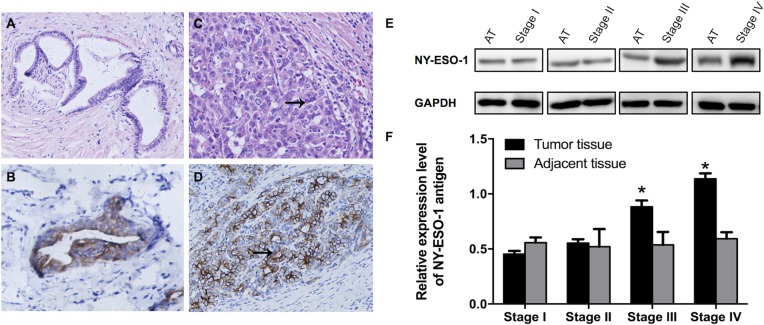
Expression of NY-ESO-1 antigen in iCCA and corresponding adjacent normal tissues (**A–D**) Representative H&E staining demonstrate the well (A) and poorly (C) differentiated iCCA tissues; Immunohistochemical micrographs show the expression of NY-ESO-1 in well (B) and poorly (D) differentiated carcinoma (Original magnification: × 400). **(E**, **F**) NYESO-1 antigen expression was significantly increased in iCCA tissue, especially in tissues with stage III and IV disease, compared with that in adjacent non-neoplastic tissues (E, F). (AT: adjacent tissues.)

Immunohistochemical staining was performed to show the NY-ESO-1 protein expression. Our results suggested that there was positive staining of NY-ESO-1 in both membrane and cytoplasm of tumor cells (Figure 1A–1D). Then, the expression of NY-ESO-1 in iCCA tissue and adjacent non-neoplastic tissues was validated using standard Western blotting procedures. The tissue specimens were obtained from all patients with iCCA. Our results indicated that the NY-ESO-1 protein expression in iCCA tissue, especially in those with higher stages from III to IV, was significantly upregulated compared with that in adjacent tissues (*p <* 0.01) (Figure 1E, 1F).

### Correlation between serum anti-NY-ESO-1 antibody and initial symptoms of iCCA

The correlation analysis between serum NY-ESO-1 antibody and initial symptoms was performed using univariate logistic regression analysis. Our findings showed that the serum level of NY-ESO-1 antibody is closely associated to abdominal pain (crude odds ratio [cOR] 4.53, 95% CI 1.29–8.31, *p <* 0.001) and jaundice/pruritus (cOR 1.28, 95% CI 1.30–4.23, *p =* 0.02). After adjustment of related co-variables in multivariate model included age, gender, smoking, alcohol drinking, AFP, T-Bil and D-Bil, the positivity of serum anti-NY-ESO-1 antibody was correlated with abdominal pain (adjusted odds ratio [aOR] 2.66; 95% CI 1.10–4.47, *p =* 0.04) in iCCA patients. However, in multivariate model, no significant correlation was found between the serum level of NY-ESO-1 antibody and that of fever (*p =* 0.68), nausea (*p =* 0.57), emesis (*p =* 0.62), jaundice/pruritus (*p =* 0.39), anorexia (*p =* 0.55), and asymptomatic demonstration (*p =* 0.84), respectively (Table 3).

**Table 3:** The correlation between anti-NY-ESO-1 positivity and the initial symptoms of intrahepatic cholangiocarcinoma

Initial demonstration	Univariate associations		Multivariate associations	
	Crud Odd ratio	*P* value	Adjusted Odd ratio	*P* value
Fever	1.83 (0.78–2.01)	0.45	1.06 (0.69–1.43)	0.68
Abdominal pain	4.53 (1.29–8.31)	<0.001	2.66 (1.10–4.47)	0.04
Nausea	1.99 (0.61–1.56)	0.26	0.95 (0.48–1.22)	0.57
Emesis	1.29 (0.81–3.27)	0.24	0.66 (0.79–2.04)	0.62
Jaundice/pruritus	1.28 (1.30–4.23)	0.02	0.97 (0.80–3.19)	0.39
Anorexia	1.58 (0.34–5.89)	0.43	1.02 (0.46–3.37)	0.55
Asymptomatic	1.05 (0.64–5.73)	0.72	0.89 (0.48–4.41)	0.84

### Prognostic performance of serum anti-NY-ESO-1 antibody in iCCA

**Figure 2:**
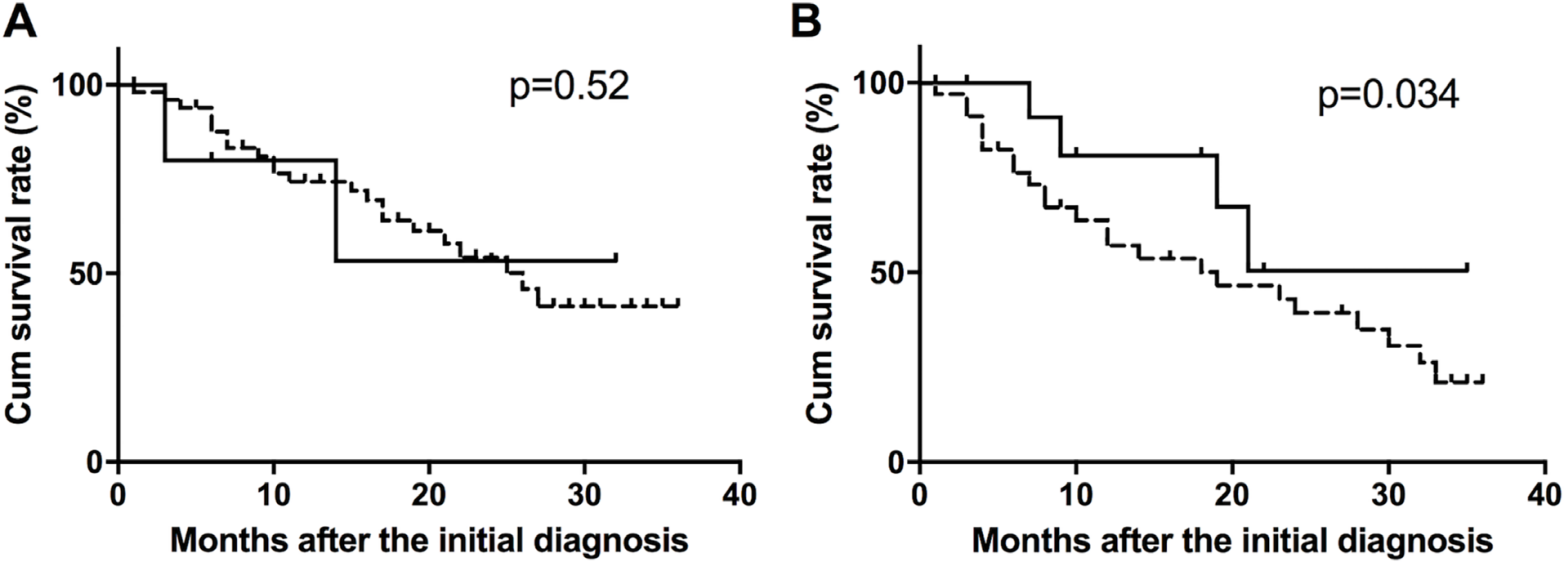
Survival curves of NY-ESO-1 antibody-positive and -negative patients The cumulative survival rate was calculated in iCCA patients with stage I + II (**A**) and stage III + IV (**B**) disease.

The effects of serum anti-NY-ESO-1 antibody on the prognosis of iCCA patients were assessed using Kaplan-Meier survival analysis with log-rank testing. In iCCA patients at stage I + II, there was no association between the cumulative survival rate and positivity of the serum anti-NY-ESO-1 antibody (*p =* 0.52) (Figure 2A). However, among the iCCA patients with stage III + IV, the cumulative survival rate was higher in serum NY-ESO-1-positive patients than in NY-ESO-1-negative patients (*p =* 0.034) (Figure 2B).

## DISCUSSION

This study documents that there is a higher frequency of serum NY-ESO-1 antibody in iCCA patients 18.4% (19/103) than in patients with CHB (3.7%, 4/108). The serum level of NY-ESO-1 antibody is correlated with pathological characteristics including tumor poorly differentiation and lymphatic metastasis and a higher cTNM stage. However, our results suggest a possibility that serum NY-ESO-1 antibody, induced in naturally occurring immune responses, might be a beneficial factor in prolonging the overall survival of iCCA.

The gene for NY-ESO-1 was localized to chromosome Xq28, a region that also includes the MAGE family that encodes protein with a characteristic of cancer or testis expression pattern [19]. The expression of NY-ESO-1 mRNA was revealed in 20–30% of esophageal cancer, melanomas, advanced prostate cancer, hepatocellular carcinoma, epithelial ovarian cancer, breast tumors, and bladder cancers [10, 14, 20, 21]. The NY-ESO-1 gene codes for an 18-kDa immunogenic testicular antigen [22]. Previous studies have suggested that the NY-ESO-1 product appears to be the most immunogenic, eliciting both humoral and cellular immunological response in a high percentage of patients with advanced NY-ESO-1-expressing tumors [12, 13, 22, 23].

NY-ESO-1, as the most immunogenic CT antigen known thus far, is widely expressed and is considered a potential target molecule for cancer vaccines in a multitude of malignancies as described above [24]. The NY-ESO-1-induced humoral immune response was lower in early-stage tumor (stage I and II) than in those with higher stage (stage III and IV) in, for instance, gastric cancer, making the NY-ESO-1 antibody insufficient for the early prediction [9]. However, as NY-ESO-1 protein is expressed in only tumor tissues and the NY-ESO-1 antibody was strictly developed by the NY-ESO-1 antigen in tumor tissue, extremely highly specific NY-ESO-1 humoral immune responses were detected in NY-ESO-1-expressing cancer patients [13]. This was also confirmed by this study in which the frequencies of antibody against NY-ESO-1 were only 3.7% in non-neoplastic disease such as CHB.

The role of antibody against CT family antigens in predicting the survival of tumor patients is controversial. In ovarian cancer, antibodies against any of the MAGE family antigens (including MAGE-A1, MAGE-A4, MAGE-A3 and MAGE-A10) were correlated with a lower survival rate [25]. On the other hand, the overall survival of patients with antibodies against CT antigens, including NY-ESO-1 and GAGED2a, was prolonged in lung cancer patients [26]. However, the current opinions are inclined to consider CT antigen-induced humoral immunity as correlating with a favorable prognosis in tumors [26, 27]. Our results also indicated that, in patients with iCCA, the cumulative survival rate was significantly higher in serum NY-ESO-1-positive patients than in serum NY-ESO-1-negative patients. These findings revealed that NY-ESO-1 related to humoral immune responses might play an important role in restraining the progression of tumors.

In summary, this study revealed that the serum level of anti-NY-ESO-1 antibody was detected as higher in patients with iCCA than in control subjects and was positively correlated with tumor differentiation, lymphatic metastasis and cTNM stage. NY-ESO-1 antibody might be a useful tumor marker and prognostic factor in iCCA. However, the present findings should be carefully interpreted and further evaluated by different patient cohorts.

## MATERIALS AND METHODS

### Patients and sample collection

Prior written and informed consent were obtained from all patients. The research protocols were approved by the ethics review board of the First Affiliated Hospital of Anhui Medical University (AHEC2016-103), and all methods were performed in accordance with the relevant ethical guidelines and regulations. All participants were recruited from 2002 to 2016 and the study was conducted in October 2016. In total, 150 hospitalized patients with histologically confirmed iCCA, who underwent surgical resection or chemotherapy at the Department of Gastrointestinal Surgery, the First Affiliated Hospital of Anhui Medical University, were enrolled in the study. Finally, complete follow-up was obtained in 103 (68.7%) patients at the end of this study. Additionally, 108 hospitalized patients with chronic hepatitis B (CHB) matched for age and sex were also recruited as controls in this study.

The serum samples were collected and stored as surplus samples after routine blood tests for patients with iCCA and as part of a routine examination for control subjects. The flash-frozen tissue specimens of tumor and adjacent tissues were obtained from iCCA patients and were stored at –80°C. Finally, the relevant patient databases were retrieved to collect medical information, including blood test results, tumor stage, histological type, invasion location, and lymphatic metastasis, which were obtained from pathological examinations and/or radiographic findings.

### Enzyme-linked immunosorbent assay (ELISA)

Anti-NY-ESO-1 antibodies were detected by ELISA. Polystyrene plates (96-well) were coated and incubated overnight at 4°C with 100 μL/well of recombinant human protein NY-ESO-1 (1 μg/ml) in phosphate-buffered saline (PBS) on the test half and PBS alone on the control half. The coated plates were washed 3 times with PBS containing 0.1% Tween 20 (PBS-T) and were blocked for 2 h at room temperature with 200 μL of 1% BSA in PBS. After washing 3 times again with PBS-T, the wells were incubated for 1 h at room temperature with 100 μL of patient serum (diluted 1:100 in the blocking buffer). Next, after extensive washing, Horseradish-Peroxidase goat anti-human IgG/polyclonal (Sigma-Aldrich, St. Louis, MO) was diluted in 1% BSA in PBS and was added to the wells as a secondary antibody, and the plates were incubated for 1 h at room temperature. The plates were washed 3 times with PBS-T; the bound peroxidase was then revealed with 100 μL of O-phenylenediamine dihydrochloride, and the signal development was stopped with 0.2 M H_2_SO_4_ incubation for 5 min. Absorbance was measured at 492 nm using a microplate reader. The data are presented as the mean optical density (OD) adjusted for background (wells coated with PBS alone). A cut-off value was defined as the mean optical density (OD) ± 3SD of normal human sera. Each serum sample was analyzed in triplicate. Ovalbumin (OVA, Sigma Aldrich) was employed as a control protein [9].

### Immunohistochemistry

Four-micron thick formalin-fixed, paraffin-embedded tissue sections were used. The sections were deparaffinized in xylene and rehydrated in graded alcohols (100%, 95% and 85%). After heat-induced epitope retrieval by retrieval solution (10 mM citrate buffer, pH 6.0) for 30 min, endogenous peroxidase was blocked using the Blocking Kit (Zhongshan Biotechnology, Beijing, China). Slides were then incubated with the primary antibodies against NY-ESO-1 (1:100 dilution, Invitrogen/Life Technologies, NY) at room temperature for 2 h. A horseradish peroxidase/3,3′-diaminobenzidine polymer-based detection system (DAKO; Envision+ System, CA) was utilized for detection of the NY-ESO-1 expression. NY-ESO-1 staining was defined as positive when the nuclear and/or cytoplasmic staining was detected.

### Western blotting analysis

The frozen tissue specimens were homogenized, lysed with pre-cooled RIPA lysis solution for 50 min at 4°C and then centrifuged at 12,000 g/min for 5 min for protein extraction. The concentration of the total protein in the supernatant was detected using the BCA-Protein Assay Kit (Thermo Scientific, USA). The total proteins were analyzed using SDS-PAGE electrophoresis and then were transferred to polyvinylidene difluoride (PVDF) membranes by an electroblot apparatus. After blocking with defatted milk for 1 h at 25°C, the primary rabbit anti-NY-ESO-1/polyclonal antibodies (1:1000, Abcam, Cambridge, MA) and mouse anti-GAPDH/polyclonal (1:5000, Abcam, Cambridge, MA) were added and incubated on a shaker overnight at 4°C. After rinsing with PBS (10 min × 3), HRP-labeled secondary rabbit or mouse IgG antibodies/polyclonal (1:5000, Abcam, Cambridge, MA) were added and incubated at room temperature for 1 h. The protein was determined by the enhanced chemiluminescence (ECL) detection system. Imaging signals of protein bands were acquired and analyzed using Image Lab Software (Bio-rad, Richmond, CA). The relative expression of NY-ESO-1 protein was defined as the ratio of gray values between the target protein and GADPH.

### Statistical analysis

All of the data were analyzed using SPSS 23.0 software (SPSS, USA). The distributions of the NY-ESO-1 antibody in different groups were analyzed by χ^2^ test. The effect of NY-ESO-1 antibody on the cumulative survival rate was plotted using Kaplan-Meier curves and log-rank testing. Values of *p <* 0.05 were considered statistically significant.
